# Human Lipoxygenase Pathway Gene Variation and Association with Markers of Subclinical Atherosclerosis in the Diabetes Heart Study

**DOI:** 10.1155/2010/170153

**Published:** 2010-05-31

**Authors:** Kathryn P. Burdon, Megan E. Rudock, Allison B. Lehtinen, Carl D. Langefeld, Donald W. Bowden, Thomas C. Register, Yongmei Liu, Barry I. Freedman, J. Jeffrey Carr, Catherine C. Hedrick, Stephen S. Rich

**Affiliations:** ^1^Department of Biochemistry, Wake Forest University School of Medicine, Winston-Salem, NC 27157, USA; ^2^Center for Human Genomics, Wake Forest University School of Medicine, Winston-Salem, NC 27157, USA; ^3^Division of Public Health Sciences, Wake Forest University School of Medicine, Winston-Salem, NC 27157, USA; ^4^Department of Internal Medicine, Wake Forest University School of Medicine, Winston-Salem, NC 27157, USA; ^5^Department of Pathology, Wake Forest University School of Medicine, Winston-Salem, NC 27157, USA; ^6^Division of Radiological Sciences, Wake Forest University School of Medicine, Winston-Salem, NC 27157, USA; ^7^Division of Inflammation Biology, La Jolla Institute of Allergy & Immunology, La Jolla, CA 92037, USA; ^8^Department of Public Health Sciences and Center for Public Health Genomics, University of Virginia, P.O. Box 800717, Charlottesville, VA 22908-0717, USA

## Abstract

*Aims*. Genes of the 5-lipoxygenase pathway are compelling candidates for atherosclerosis. We hypothesize that polymorphisms in *ALOX12, ALOX15, ALOX5*, and *ALOX5AP* genes are associated with subclinical atherosclerosis in multiple vascular beds. *Methods*. Families with two or more siblings with type 2 diabetes and their nondiabetic siblings were studied as part of the Diabetes Heart Study (DHS). European American diabetic (*n* = 828) and nondiabetic (*n* = 170) siblings were genotyped for SNPs in the *ALOX12, ALOX15, ALOX5*, and *ALOX5AP* genes. Subclinical measures of atherosclerosis (IMT, coronary (CorCP), carotid (CarCP) and aortic (AorCP) calcified plaque) were obtained. *Results*. Associations were observed between *ALOX12* with CorCP, *ALOX5* with CorCP, AorCP, and IMT, and *ALOX5AP* with CorCP and CarCP, independent of known epidemiologic risk factors. Further, lipoxygenase pathway SNPs that were associated with measures of atherosclerosis were associated with markers of inflammation (CRP, ICAM-1) and calcification (MGP). *Conclusions*. Polymorphisms within *ALOX12, ALOX5*, and *ALOX5AP* are genetically associated with subclinical atherosclerosis and with biomarkers of disease in families with type 2 diabetes. These results suggest that variants in lipoxygenase pathway genes may have pleiotropic effects on multiple components that determine risk of cardiovascular disease.

## 1. Introduction

Atherosclerosis is thought to be the result of chronic inflammation of the artery wall although the pathways and factors that initiate and modulate the inflammatory response in atherosclerosis have yet to be completely resolved [1]. Metabolites of arachidonic acid are strong candidates that are recognized for their inflammatory properties. The mouse 5-LO gene, *ALOX5*, has been shown to contribute to the development of atherosclerosis [2]. Variants in the human homologue (*ALOX5*) are associated with carotid artery intima-media thickness (IMT) [3]. FLAP (5-lipoxygenase activating protein), encoded by the *ALOX5AP* gene, likely acts as an arachidonic acid-binding and transfer protein to facilitate 5LO activity [4]. Single SNPs and haplotypes of *ALOX5AP* have been associated with myocardial infarction in multiple populations [5–7]. 

Human 12-lipoxygenase (encoded by *ALOX12*) and 15-lipoxygenase (encoded by *ALOX15*) have been localized to atherosclerotic plaques, suggesting that 12/15LO activity is involved in the development of atherosclerosis [8–10]. Overexpression of human 15-LO in mouse vascular endothelial cells increased markers of atherosclerosis [11]. Human aortic endothelial cells cultured in chronically high glucose levels results in elevated levels of 12S-HETE, suggesting activation of lipoxygenase pathways [12, 13]. 

The current research was motivated by the role of lipoxygenase pathway gene products in inflammation, initiation/progression of atherosclerosis, and their modulation of expression by glucose. The *ALOX12*, *ALOX15*,* ALOX5,* and *ALOX5AP* genes represent strong candidates for atherosclerosis risk, especially in the context of type 2 diabetes. We have assessed genetic variants (SNPs) in lipoxygenase pathway genes for evidence of association with markers of subclinical atherosclerosis (intima-media wall thickness [IMT], carotid artery calcified plaque [CarCP], coronary artery calcified plaque [CorCP], and aortic calcified plaque [AorCP]) and biomarkers (e.g., ICAM-1, E-selectin, CRP) in participants of the Diabetes Heart Study (DHS). 

## 2. Patients and Methods

### 2.1. Participants

Recruitment and phenotyping of Diabetes Heart Study (DHS) participants have been previously described [14–16]. Siblings concordant for type 2 diabetes were recruited if they had no evidence of renal insufficiency, as were all available nondiabetic siblings. Participant examinations were conducted in the General Clinical Research Center of the Wake Forest University School of Medicine, and included interviews for medical history, medication use and health behaviors, anthropometry, resting blood pressure, a fasting blood sampling, and a spot urine collection. Only Caucasian participants were included in this report, due to small sample size and limited statistical power in the African-American cohort. All study protocols were approved by the Institutional Review Board of Wake Forest University School of Medicine. All participants provided written informed consent.

### 2.2. Measurements

Intima-media thickness (IMT) of the common carotid artery was measured by high-resolution B-mode ultrasonography with a 7.5-MHz transducer and a Biosound Esaote (AU5) ultrasound machine [14]. The mean of up to 20 common carotid IMT estimates was used as the phenotypic. Calcified plaque was measured in the carotid and coronary arteries and the aorta using single and multidetector CT systems that employ a standardized protocol which includes CT scanner phantom testing based on those currently implemented in the National Heart Lung and Blood Institute's (NHLBI) Multiethnic Study of Atherosclerosis (MESA) studies [15, 17].

### 2.3. Molecular Genetics

Total genomic DNA was purified from whole blood samples obtained from subjects using PUREGENE DNA isolation kit (Gentra, Inc., Minneapolis, MN). DNA was quantitated using standardized fluorometric readings on a Hoefer DyNA Quant 200 fluorometer (Hoefer Pharmacia Biotech Inc., San Francisco, CA). Each sample was diluted to a final concentration of 5 ng/*μ*L. Single nucleotide polymorphisms (SNPs) were identified for *ALOX12* (17p13.1), *ALOX15* (17p13.3), *ALOX5* (10q11.2), and *ALOX5AP* (13q12) and were chosen for genotyping in order to provide coverage of linkage disequilibrium (LD) blocks using the Program in Genome Applications (PGA, University of Washington) with *r* ^2^ < 0.8 and minor allele frequency (MAF) greater than 5%. As much of the HapMap data were not available at the time of the study, relatively few SNPs were available for each locus that passed the selection criteria and were suitable for the genotyping platform. All SNP genotypes were determined using a MassARRAY SNP genotyping system (Sequenom, Inc., San Diego, CA). 

### 2.4. Statistical Analyses

The sample means, standard deviations (SD), and medians were computed on continuous variables, while proportions were determined for discrete variables in those DHS participants who contributed to the genetic analyses. Variables were transformed, when required, to meet normality assumptions. A series of generalized estimating equations (GEE) [18] assuming exchangeable correlation structure and using the empirical estimate of the variance (to adjust for familial correlation induced by selection of related individuals in sibships) was used for assessment of the effects of diabetes status on clinical variables. 

### 2.5. Statistical Genetic Analyses

Allele and genotype frequencies were determined with unrelated subjects (a single member from each family) and tested for departure from Hardy-Weinberg proportions using a chi-square test (deviations with *P <* .001 were considered significant). Association between each individual SNP and each phenotype was determined using the quantitative pedigree disequilibrium test (QPDT) [19]. The QPDT method reduces the effect of undetected stratification bias as transmission, rather than frequency of specific SNP alleles, is used in estimating the association between SNP and phenotype. All association analyses were conducted with adjustment for known epidemiologic risk factors of atherosclerosis (age, gender, diabetes status, smoking, BMI, use of aspirin, estrogen (women only), and lipid-lowering and hypertension medications). 

For any SNP that exhibited a significant association with an atherosclerosis phenotype, additional SNPs within the LD block were examined (when available). QPDT SNP haplotypes were determined using the EM algorithm. QPDT analyses were applied to these haplotypes, adjusting for the same risk factors as in the single SNP analyses. Genotype-specific means, medians, and number of subjects within each genotypic class and for each measure of atherosclerosis were computed under a specific genetic model. Analyses of biomarkers (e.g., ICAM-1, E-selectin, CRP) were performed using the same analytic strategy.

## 3. Results

### 3.1. Patient Characteristics

DNA was collected from 998 European-American subjects from 368 families. Demographic characteristics of participants are shown in Table 1. The age at ascertainment of diabetic cases (62.0 ± 9.3 years) was slightly older than the nondiabetic siblings (59.5 ± 10.0 years). Duration of diabetes was 10.4 ± 7.1 years, reflecting a relatively early age at onset of type 2 diabetes (~52 years). BMI was higher in diabetic siblings (32.4 ± 6.7 kg/m^2^) than in nondiabetic siblings (28.9 ± 5.2 kg/m^2^). The nondiabetic subjects were, on average, overweight (as defined by a BMI between 25–29.9 kg/m^2^) and approaching obese. There were few differences in lipid (total cholesterol, HDL, LDL) profiles between diabetic and nondiabetic subjects, although lipid lowering agents were used more commonly in the diabetic subjects (45.4%) than in the nondiabetic subjects (27.8%). Current and past smoking was highly prevalent in both diabetic (59.4%) and nondiabetic (57.4%) participants. 

Cardiovascular characteristics of the DHS participants are shown in Table 2. Prevalence of CVD and extent of atherosclerosis (CorCP, CarCP, AorCP, IMT) varied by location (vascular bed) and diabetes. Diabetic participants had greater prevalence of all events/procedures (heart attack, angina, stroke, CABG, angioplasty) than nondiabetic siblings. Diabetic participants had significantly more calcified plaque in the coronary (1425 ± 29 versus 551 ± 13, mean ± stderr), carotid (374 ± 25 versus 168 ± 35), and aorta (3995 ± 166 versus 2342 ± 309) than nondiabetic siblings; however, there was no significant difference in IMT with respect to diabetes status (0.68 ± 0.01 in diabetics, 0.64 ± 0.01 in nondiabetics).

### 3.2. Lipoxygenase Pathway Gene SNPs

Allele and genotype frequencies of SNPs in *ALOX12*,* ALOX15*, *ALOX5, *and* ALOX5AP *were assessed for deviation from Hardy-Weinberg expectations in unrelated probands (see Supplementary Table 1 in Supplementary Material available online at doi:10.1155/2010/170153). No SNP exhibited significant (*P <* .001) deviation from Hardy-Weinberg equilibrium. All SNPs were maintained in subsequent analyses. The distribution of SNPs provided coverage within the block structure of the genes, as well as providing additional coverage (either 3′ or 5′) of the regions adjacent to the lipoxygenase pathway genes. 

### 3.3. Lipoxygenase Pathway Genes and Coronary Calcified Plaque (CorCP)

None of the 17 tagging SNPs in the lipoxygenase pathway genes were significantly (*P <* .05) associated with CorCP (Table 3). 

In exploratory analyses, four SNPs exhibited suggestive association (*P <* .10) with CorCP, one in *ALOX12* (rs2271316, *P = *.061, 3′ of the gene), one in *ALOX5* (rs2115819, *P = *.090, intron 3), and two in *ALOX5AP* (rs9506352, *P = *.097, intron 2; rs4769060, *P = *.073, intron 4). In *ALOX12*, another SNP near rs2271316 was identified (rs1042357, exon 8) and was genotyped. This SNP (rs1042357) is only 10 kb from rs2271316. The association of rs1042357 with variation in CorCP was not significant (*P = *.211) and the two-SNP haplotype was also not significant (*P = *.157). Hence, the *ALOX12* marginal association was not confirmed. In *ALOX5*, two additional SNPs were identified (rs1369214, intron 3; rs11239524, intron 4) and were genotyped. These two SNPs bound the *ALOX5* marginally associated SNP (rs2115819, intron 3). The *ALOX5* SNP rs1369214 is only 360 bp from rs2115819, while the *ALOX5* SNP rs11239524 is ~11 kb from *ALOX5* SNP rs2115819. The association between CorCP with rs1369214 was similar in magnitude (*P = *.098), while the association between CorCP with rs11239524 was much stronger (*P = *.027). Two- and three-SNP haplotypes were not more strongly associated than either the original SNP (rs2115819 in intron 3) or the SNP in intron 4 (rs11239524), suggesting that there may be an effect of *ALOX5* on variation of CorCP in intron 4 worthy of further examination. In *ALOX5AP*, two SNPs were identified as having a marginal effect on variation in CorCP (rs9506352 in intron 2 and rs4769060 in intron 4). Since the distance between these two SNPs is ~17 kb, three additional SNPs (rs4769874 in intron 3, rs9315048 in intron 3, and rs12019512 in intron 4) were genotyped in these samples. The three SNPs were not significantly associated with CorCP, and analyses of two-SNP haplotypes failed to increase evidence of association. Thus, the exploratory genotyping and data analyses for *ALOX5AP* SNP did not increase evidence of association with CorCP.

### 3.4. Lipoxygenase Pathway Genes and Carotid Calcified Plaque (CarCP)

No SNPs in the lipoxygenase pathway genes were significantly (*P <* .05) associated with CarCP (Table 3). 

In exploratory analyses, one SNP in *ALOX5AP* (rs10507391, *P = *.085, intron 1) exhibited suggestive association (*P <* .10). Two tagging SNPs adjacent to rs10507391 (rs4769055 and rs9551960) span a 7 kb region in intron 1 of *ALOX5AP*. Two-SNP haplotype analyses identified the rs10507391-rs9551960 haplotype as significantly associated with variation in CarCP (*P = *.002). The rs10507391-rs9551960-rs9506352 three-SNP haplotype was also strongly associated with CarCP (*P = *.003), while the rs4769055-rs10507391-rs9551960 haplotype was not associated (*P =* 374). These data suggest that the *ALOX5AP* effect on CarCP may reside between rs10507391 (intron 1) and rs9506352 (intron 2). No additional genotyping of other *ALOX5AP* SNPs was performed.

### 3.5. Lipoxygenase Pathway Genes and Aortic Calcified Plaque (AorCP)

No tagging SNP in the four lipoxygenase pathway genes were significantly associated (*P <* .05) with AorCP (Table 3). 

In exploratory analyses, one *ALOX5* SNP (rs2115819 in intron 3) exhibited the strongest (*P = *.108) association. The rs2115819 SNP was also associated with variation in CorCP. Two additional SNPs near the *ALOX5* rs2115819 SNP were identified for examination. Genotyping these two adjacent SNPs in *ALOX5* (rs1369214, intron 3; rs11239524, intron 4) failed to provide evidence of association, either in analyses of single SNPs or two- and three-SNP haplotypes. Thus, the marginal association with AorCP was not confirmed.

### 3.6. Lipoxygenase Pathway Genes and Intima-Media Thickness (IMT)

None of the tagging SNPs in the lipoxygenase pathway genes were significantly (*P <* .05) associated with IMT (Table 3). 

In exploratory analyses, one *ALOX5* SNP (rs3780906 in intron 6) was marginally associated (*P = *.090) with IMT. Two additional *ALOX5* SNPs (rs3780901 in intron 4 and rs1059696 in intron 7) were identified that were near the *ALOX5* rs3780901 SNP. These SNPs were genotyped and tested for association. Neither bordering SNP was significantly associated with IMT (rs3780901, *P = *.871; rs1059696, *P = *.746). The distance spanned in *ALOX5* by the three SNPs was 17 kb. The two-SNP haplotype formed by rs3780901-rs3780906 (12 kb between intron 4 and intron 6) was significantly associated with IMT (*P = *.047). Further, the three-SNP haplotype (rs3780901-rs3780906-rs1059696) was strongly associated with IMT (*P = *.019). Thus, this region in *ALOX5* may be worthy of further examination for association with IMT.

### 3.7. Phenotypic Effect of Lipoxygenase Pathway Gene Variants on Measures of Atherosclerosis

Genotype-specific means, medians, and number of subjects within each genotypic class and for each measure of subclinical atherosclerosis are presented in Table 4. Although no statistically significant effects were observed based upon comparisons of genotypic means (due to large variances of measured phenotypes and long-tailed phenotypic distributions), there were interesting trends in comparison of genotypic medians.

For the *ALOX5* rs2115819 SNP with CorCP and AorCP, the genotype-specific means suggested an inheritance pattern consistent with a dominant effect of allele “1”, with the group mean (±SE) for the combined (1/1 and 2/1) genotypes having significantly less CorCP (1127 ± 95) and AorCP (3026 ± 183) than the 2/2 genotype (1540 ± 135 and 5318 ± 540, resp.). The “dominant” pattern is also seen for the *ALOX12* rs2271316 SNP [1/1 and 2/1 genotypes having significantly less CorCP (1097 ± 97) than the 2/2 genotype (1423 ± 216)] and for the *ALOX5AP* rs9506352 SNP [1/1 and 2/1 genotypes having significantly less CorCP (1145 ± 87) than the 2/2 genotype (1672 ± 465)]. However, for the *ALOX5AP* rs4769060 SNP, the effect on CorCP appears more consistent with additivity, with the 1/1 genotype having least CorCP (996 ± 114), the 2/1 genotype having medium CorCP (1244 ± 132), and the 2/2 genotype having the greatest CorCP (1404 ± 247). 

For CarCP, the *ALOX5AP* rs10507391 SNP exhibited an additive pattern. The 1/1 genotype had greatest CarCP (360 ± 37), the 2/1 genotype had intermediate CarCP (313 ± 36), and the 2/2 genotype had the least CarCP (216 ± 43). The effect of the *ALOX5* rs3780906 SNP on IMT was also “additive,” with the 1/1 genotype having the least IMT (0.666 ± 0.006), the 2/1 genotype having intermediate IMT (0.675 ± 0.007), and the 2/2 genotype having the greatest IMT (0.687 ± 0.016).

### 3.8. Functional Support for SNP Effects

The associations of lipoxygenase pathway gene variants (*ALOX* SNPs) with measures of atherosclerosis are indirect. The potential roles of these SNPs on mechanisms of atherosclerosis were explored by estimating the effect of each associated SNP in a select panel of biomarkers. The biomarkers evaluated in this population include adiponectin, leptin, ICAM-1, VCAM-1, E-selectin IL-6, CRP, MGP, and MCP-1 (Table 5).

Four lipoxygenase pathway variants contributed to variation in CorCP—one in *ALOX5* (rs2115819), one in *ALOX12* (rs2271316), and two in *ALOX5AP* (rs9506352 and rs4769060). The two *ALOX5AP* SNPs exhibited significant associations with levels of adiponectin (rs9506352, *P = *.044; rs4769060, *P = *.007), CRP (rs9506352, *P = *.028; rs4769060, *P = *.002); and MGP (rs9506352, *P = *.003; rs4769060, *P = *.037). Levels of MGP varied in a manner consistent with an additive gene effect, based upon the observed genotypic means [rs9506352 GG (9.06), GA (8.08), AA (7.64); rs4769060 AA (8.89), AG (8.41), GG (7.88)]. The *ALOX12* SNP also was associated with CRP level (*P = *.036), but also significantly associated with ICAM-1 level (*P = *.032) in a recessive pattern [rs2271316 GG (295.7), GC (268.3), CC (266.0)]. The *ALOX5* SNP (rs2115819) was associated with both CorCP and AorCP. This SNP was also associated with ICAM-1 (*P = *.037) and E-selectin (*P = *.0001); however, the genotypic means did not fit a classical single gene model, as the levels of both ICAM-1 and E-selectin for the heterozygote (T/C) class were greater than the means for the homozygote classes. Nonetheless, these data suggest that lipoxygenase pathway variants that are associated with CorCP affect markers of inflammation (CRP, ICAM-1) as well as arterial calcification (MGP).

One SNP in *ALOX5AP* (rs10507391) was associated with CarCP in this population. The *ALOX5AP* SNP identified a three-SNP haplotype (rs10507391-rs9551960-rs9506352) that was highly associated with CarCP. Each of these three individual SNPs was significantly (*P <* .05) associated with MGP level [rs10507391 AA (7.87), AT (8.63), TT (9.83); rs9551960 GG (7.69), GA (8.56), AA (9.64); rs9506352 GG (9.06), GA (8.08), AA (7.64)] as was the haplotype.

 A single SNP in *ALOX5* (rs3780906) was associated with variation in IMT, with a three-SNP haplotype (rs3780901-rs3780906-rs1059696) providing strongest evidence of association. No consistent pattern of association was evident with any biomarker for the single SNP or the three-SNP haplotype.

## 4. Discussion

Type 2 diabetes is a major risk factor for cardiovascular disease (CVD), whose clinical outcomes include myocardial infarction and ischemic stroke. The principle etiologic factor for CVD is atherosclerosis which is thought to be the result of a chronic inflammatory process within a vessel wall that precipitates a cascade of events, from establishment of a fatty streak lesion to plaque formation [20]. The inflammatory process is triggered, in part, by oxidized lipids, including those of the lipoxygenase pathway. Recent evidence has demonstrated that lipoxygenases have two basic functions, (a) membrane modification by peroxidation and (b) lipid mediator signaling by G protein-coupled receptors [21, 22]. 

In the mouse, regulation of 12/15-LO and its pathway components (12S-HETE and 13S-HODE) in the vessel wall modulates aortic monocyte/endothelial cell interactions [23, 24], which are key early events in vascular inflammation [25]. Thus, the 12/15-LO pathway primarily affects atherosclerosis through LDL oxidation. An alternative pathway involves biosynthesis of proinflammatory leukotrienes (e.g., LTB_4_) by 5-LO (and 5-LO-activating protein, FLAP, which transfers arachidonate to 5-LO), providing access to downstream leukotrienes binding to G protein-coupled receptors. Recently, it has been shown that 5-LO pathway components are highly expressed in arterial walls in patients with atherosclerosis of the carotid and coronary arteries and the aorta [26]. 

Leukocyte-specific expression of human 15-LO has also been shown to reduce inflammation and atherosclerosis in rabbits [27, 28], most likely due to the generation of lipoxins from the dual action of 5-LO and 15-LO on arachidonic acid substrate in the leukocyte [29–31]. Studies in mice in which 15-LO was selectively expressed in endothelium indicated that endothelial-specific overexpression of 15-LO accelerated progression of atherosclerosis [12]. One plausible explanation for these differences is that endothelial cells do not possess 5-LO and are unable to directly generate lipoxins or related anti-inflammatory eicosanoids. The cellular source of lipoxygenase may be a critical determinant of atherosclerosis; hence, lipoxygenase pathways, their components, and their genetic determinants may be central to understanding the development of human atherosclerosis and CVD risk. 

Genetic factors have long been known to modulate risk of atherosclerosis and CVD [32, 33]. In studies enriched with diabetic individuals or those with CVD, the genetic contribution to variation in carotid artery IMT ranges from 42%–92% [14, 34, 35]. Significant genetic contribution to calcified plaque has also been observed, whether in the coronary arteries [36], the carotid arteries [37, 38], or the aorta [39]. 

In the Diabetes Heart Study (DHS), variation in quantitative measures of subclinical atherosclerosis (CorCP, CarCP, AorCP, and IMT) appears to be differentially influenced by variants in genes of the lipoxygenase pathway. SNPs in *ALOX5* are associated with variation in CorCP, AorCP, and IMT. SNPs in *ALOX5AP* are associated with variation in CorCP and CarCP, while SNPs in *ALOX12* are associated with variation in CorCP. These results are consistent with findings that have been emerging from cellular and mouse models of atherosclerosis. Enhanced LDL oxidation, IL12 production, and endothelial/monocyte interaction have been observed through manipulation of 12/15-LO [11, 25, 40, 41]. In mouse genetic studies, a region on mouse chromosome 6 (the site of 5-LO) was shown to be linked to atherosclerosis susceptibility [42, 43]. Later, it was demonstrated that the disruption of only one 5-LO allele significantly reduced the extent of atherosclerotic lesions at the aortic root in LDLR−/− mice [2]. 

Previously, an *ALOX5* variant, defined by the number of Sp1 binding motifs in the promoter, was shown to be associated with variation in IMT and CRP level (a marker of chronic inflammation) in a healthy population [3]. The promoter variant was also shown to interact with dietary intake of 5-LO substrates. The study population (Los Angeles Atherosclerosis Study), in addition to being “healthy,” was composed of several ethnic groups (Hispanic subjects and smokers were oversampled). The DHS, on the other hand, consists of European-American and African-American families with at least two diabetic siblings, making direct comparisons difficult. In addition, characterization of variation in the *ALOX5* gene was different (number of promoter Sp1 binding motifs versus SNPs within LD blocks across the entire *ALOX5* gene). Despite these differences in design and genetic evaluation, both studies observed that polymorphisms in *ALOX5* were associated with variation in measures of atherosclerosis (IMT in both studies; CorCP and AorCP in DHS). Unlike the Los Angeles Atherosclerosis Study, we did not detect an association between SNPs in LD blocks of *ALOX5* on ultrasensitive CRP level (data not shown). 

The mechanism for associations between genes of the lipoxygenase pathway and subclinical atherosclerosis is not clear, although it may involve chemotaxis and proliferation that is induced by the effects of leukotrienes B_4_ (LTB_4_) signaling in vascular smooth muscle cells. LTB_4_ has been detected in human carotid artery, atherosclerotic plaques and is derived from the 5-LO metabolism (via *ALOX5AP* and through G-coupled protein receptors) of arachidonic acid [21, 44]. Recently, a variant of the gene encoding LTB_4_ hydrolase (*LTA4H*), a protein in the same biological pathway as *ALOX5AP* has been shown to be associated with risk of myocardial infarction [45], further strengthening the case for a role of the lipoxygenase pathway on CVD risk.

These data suggest that there may be differential effects of genes in a common pathway on several vascular beds through diverse inflammatory mechanisms (based upon the effects of lipoxygenase pathway SNPs on subclinical atherosclerosis and on markers of inflammation (CRP, E-selectin, ICAM-1) and aspects of calcification. Recently, serum MGP levels were determined in 2 independent populations free of clinically apparent cardiovascular disease [46] and an association of circulating MGP with increasing Framingham CHD risk score was observed, as were associations of circulating MGP with HDL and other individual CHD risk factors. Further characterization of genes in the lipoxygenase pathway may provide important clues to prediction of CVD. Although variation in these genes may be associated with risk of atherosclerosis, they may also modulate the impact of other atherosclerotic risk factors (e.g., lipid levels) or factors that are independent of traditional risk factors. The current data suggest that knowledge of the genetic profile of a pathway (and, by extension, the interaction of components of the pathway) may improve the prediction of risk. The extent of improvement should be greater once the multilocus examination of the pathway components becomes feasible. In this manner, the genetic “biological network” [47] of atherosclerosis may become a reality.

## Supplementary Material

The supplementary material provides SNP information for the genes under investigation, along with *P*-values for deviations from Hardy Weinberg equilibrium.

## Figures and Tables

**Table 1:** Clinical and laboratory characteristics of Caucasian DHS participants.

	Diabetic patients (*n* = 828)	Nondiabetic patients (*n* = 170)
Age (years)	62.0 ± 9.3	59.5 ± 10.0
Female (%)	51.20%	61.80%
Diabetes duration (years)	10.4 ± 7.1	
BMI (kg/m^2^)	32.4 ± 6.7	28.9 ± 5.2
Cholesterol (mmol/L )	4.8 ± 1.1	5.0 ± 0.9
HDL (mmol/L)	1.1 ± 0.3	1.2 ± 0.4
LDL (mmol/L)	2.7 ± 0.8	2.9 ± 0.8
CRP (mg/L)	5.1 ± 8.3	4.1 ± 6.5
Smoking (current/past) (%)	59.40%	57.40%
Lipid lowering (%)	45.40%	27.80%
Estrogen (%, in women)	26.60%	34.80%
Aspirin (%)	57.60%	53.30%
Hypertension (%)	79.40%	46.50%

**Table 2:** Prevalence of CVD (%) and mean (± SD) for atherosclerosis phenotypes (CorCP, CarCP, AorCP, IMT) for Caucasian participants in the Diabetes Heart Study (DHS).

	Diabetic patients (*n* = 828)	Nondiabetic patients (*n* = 170)
Prevalent CVD		
Heart attack (%)	22.10%	8.30%
Angina (%)	20.40%	8.70%
Stroke (%)	10.40%	5.40%
CABG (%)	15.60%	7.70%
Angioplasty (%)	17.10%	5.30%
Endarterectomy (%)	2.30%	2.40%
Vascular Imaging		
Coronary calcified plaque	1425 ± 2637	551 ± 1187
Carotid calcified plaque	374 ± 726	168 ± 457
Aortic calcified plaque	3995 ± 4792	2342 ± 4033
Carotid IMT (mm)	0.68 ± 0.13	0.64 ± 0.12

**Table 3:** Significance of association tests of *ALOX12*, *ALOX15*, *ALOX5*, and *ALOX5AP *SNPs with measures of atherosclerosis, independent of epidemiologic risk factors.

			*P*-values*			
Gene	LD Block	SNP	CorCP	CarCP	AorCP	IMT
*ALOX12*		rs9904779	0.308	0.853	0.533	0.874
(1 block)	1	rs2292350	0.523	0.552	0.436	0.827
		rs2271316	0.061	0.839	0.555	0.683

*ALOX15*		rs11568061	0.654	0.334	0.142	0.415
(2 blocks)	2	rs2515889	0.628	0.413	0.371	0.245
	1	rs2619112	0.872	0.117	0.425	0.296

*ALOX5*	1	rs745986	0.183	0.743	0.157	0.478
(8 blocks)	2	rs2115819	0.09	0.553	0.108	0.286
	3	rs892691	0.67	0.192	0.74	0.849
	4	rs3780906	0.603	0.203	0.541	0.09
	7	rs2291427	0.114	0.964	0.423	0.336

*ALOX5AP*		rs17244974	0.251	0.572	0.492	0.173
(5 blocks)	1	rs4769055	0.201	0.805	0.242	0.376
	2	rs10507391	0.288	0.085	0.648	0.473
	3	rs9551960	0.591	0.136	0.174	0.591
	4	rs9506352	0.097	0.369	0.188	0.511
	5	rs4769060	0.073	0.378	0.456	0.299

*Analyses performed using the quantitative pedigree disequilibrium test (QPDT); *P*-values adjusted for age, gender, diabetes status, smoking status, lipid lowering medication use, BMI, estrogen use, aspirin use, and hypertension medication use.

**Table 4:** Genotypic means (± SD) of lipoxygenase variants in *ALOX5*, *ALOX5AP,* and *ALOX12* with atherosclerosis phenotypes.

Gene	SNP	1/1	1/2	2/2
CorCP				

*ALOX5 *	rs2115819	1007 ± 2088	1211 ± 2489	1540 ± 2893
		181	264	454
		(247)	(352)	(145)
*ALOX12 *	rs2271316	1065 ± 1639	1109 ± 2299	1423 ± 3155
		308	223	262
		(134)	(341)	(208)
*ALOX5AP *	rs9506352	1101 ± 2142	1188 ± 2416	1672 ± 3721
		265	229	254
		(331)	(332)	(64)
	rs4769060	996 ± 1767	1244 ± 2565	1404 ± 3086
		242	264	161
		(238)	(377)	(130)

CarCP				

*ALOX5AP *	rs10507391	360 ± 702	313 ± 694	216 ± 405
		60	71	5
		(358)	(368)	(89)

AorCP				

*ALOX5 *	rs2115819	3208 ± 4350	2908 ± 3791	5318 ± 5563
		1256	1338	3158
		(188)	(291)	(105)

IMT				

*ALOX5 *	rs3780906	0.666 ± 0.127	0.675 ± 0.129	0.687 ± 0.139
		0.649	0.654	0.645
		(436)	(348)	(75)

*The mean phenotypic value for each genotype (1/1, 2/1, 2/2) is provided with the median and the number of individuals (*n*) with each genotype included in the analysis; numbers vary based upon those with genotypic and phenotypic data.

**Table 5:** Association of lipoxygenase variants in *ALOX5*, *ALOX5AP,* and *ALOX12* with mean levels of biomarker phenotypes.

Gene	SNP		ICAM-1	CRP	IL-6	E-selectin	Leptin	Adiponectin	MCP-1	MGP
*ALOX5*	rs2115819	TT	269.6	0.62	4.34	47.7	15.2	11.82	460.6	8.44
		TC	294.8	0.66	4.58	79.7	17.9	11.01	446.5	8.47
		CC	236.4	0.64	4.16	54.2	13.6	11.05	468.9	8.53
	rs3780906	GG	269.8	0.66	3.95	67.6	13.6	11.13	444.7	8.48
		GA	286.7	0.65	4.77	59.6	21.2	11.53	459.5	8.54
		AA	228.9	0.56	4.5	51.9	6.6	11.8	505.3	7.87

*ALOX12*	rs2271316	GG	295.7	0.43	3.14	58.4	25.5	12.33	439.4	8.5
	(17947)	GC	268.3	0.75	4.68	59.3	15.7	11.03	465.3	8.59
		CC	266	0.56	4.31	67.3	13.4	11.01	429.8	8.09

*ALOX5AP*	rs10507391	AA	272.6	0.67	4.96	57.6	20.5	11.46	450.8	7.87
	(4431)	AT	262.2	0.6	3.9	63.8	13	11.77	459.3	8.62
		TT	290.1	0.7	4.82	77.1	15.7	9.52	459	9.83
	rs9506352	GG	273.2	0.69	4.3	68.7	12.9	10.69	451.7	9.06
	(13151)	GA	267.4	0.55	4.32	59.8	15.4	11.93	457	8.08
		AA	281	0.76	4.22	58.8	26	12.35	472.3	7.64
	rs4769060	AA	290.6	0.71	4.76	70.2	12.6	9.88	466.7	8.89
	(30231)	AG	260.9	0.57	4.23	61.1	14.6	12.1	450.9	8.41
		GG	278.1	0.74	4.4	63.2	22.6	11.75	443.7	7.88
