# Supplementary material for: Human Lipoxygenase Pathway Gene Variation and Association with Markers of Subclinical Atherosclerosis in the Diabetes Heart Study
Source: Mediators Inflamm. 2010 May 31;2010:170153. doi: 10.1155/2010/170153 (PMC2878676; doi:10.1155/2010/170153)
Supplement: Supplementary file 1 — The supplementary material provides SNP information for the genes under investigation, along with P-values for deviations from Hardy Weinberg equilibrium. [file 170153.f1.pdf]

Supplementary Table 1. Gene Nomenclature, LD block and physical location, alleles, minor allele frequencies, and P-values for deviation from Hardy Weinberg equilibrium (HWE).

Gene	LD Block	dbSNP	PGA*	Site	Alleles 1 / 2	Allele 2 Frequency	HWE P-value
ALOX12	-	rs9904779	1158	5’ of gene	G/C	0.41	0.825
(1 block)	1	rs2292350	4216	Intron 2	G/A	0.41	0.045
	-	rs2271316	17947	3’ of gene	G/C	0.42	0.737
ALOX15	-	rs11568061	190	5’ of gene	C/T	0.19	0.111
(2 blocks)	2	rs2515889	9429	Intron 8	G/C	0.12	0.073
	1	rs2619112	11256	Intron 2	C/T**	0.44	0.191
ALOX5	1	rs745986	-	Intron 2	A/G	0.18	0.031
(8 blocks)	2	rs2115819	-	Intron 3	T/C	0.45	1
	3	rs892691	-	Intron 4	G/A	0.29	0.313
	4	rs3780906	-	Intron 6	G/A	0.28	0.085
	7	rs2291427	-	Intron 8	G/A	0.34	0.129
ALOX5AP	-	rs17222919	660	5’ of gene	T/G	0.16	0.7
(5 blocks)	1	rs4769055	2161	Intron 1	A/C	0.31	0.223
	2	rs10507391	4431	Intron 1	A/T	0.33	0.338
	3	rs9551960	9276	Intron 1	G/A	0.42	0.007
	4	rs9506352	13151	Intron 2	G/A	0.32	0.465
	5	rs4769060	30231	Intron 4	A/G	0.43	0.915

* Program in Genomic Application (PGA) identifier (http://pga.gs.washington.edu/)

** PGA site polymorphism: C/T; dbSNP and HapMap site polymorphism: A/G
